# Radial Pulse Wave Signals Combined with Ba-PWV for the Risk Prediction of Hypertension and the Monitoring of Its Accompanying Metabolic Risk Factors

**DOI:** 10.1155/2020/3926851

**Published:** 2020-04-29

**Authors:** Zhen Qi, Zhi-Yue Zhao, Jia-Tuo Xu, Li-Ping Zhu, Yu Zhang, Yi-Min Bao, Zhi-Feng Zhang

**Affiliations:** ^1^Shanghai Geriatric Institute of Chinese Medicine, Shanghai University of Traditional Chinese Medicine, 365 South Xiangyang Road, Shanghai 200031, China; ^2^Basic Medical College, Shanghai University of Traditional Chinese Medicine, 1200 Cailun Road, Shanghai 201203, China; ^3^Physical Examination Center, The First People's Hospital of Taicang Affiliated to Suzhou University, 58 South Changsheng Road, Taicang 215400, China; ^4^Department of Cerebral Surgery, The First People's Hospital of Taicang Affiliated to Suzhou University, 58 South Changsheng Road, Taicang 215400, China

## Abstract

Our aim was to study whether radial pulse wave signals can improve the risk prediction of incident hypertension and are associated with its concomitant metabolic risk factors beyond the traditional cardiovascular risk factor Ba-PWV. By enrolling 523 Chinese subjects in this study, linear and stepwise regression analysis was performed to assess the association of radial artery pulse wave signals and Ba-PWV with blood pressure and its related metabolic risk factors such as fasting plasma glucose (FPG), total cholesterol (TC), triglyceride (TG), high-density lipoprotein cholesterol (HDL-C), low-density lipoprotein cholesterol (LDL-C), and uric acid (UA). The area under the receiver-operating characteristic curve (AUC), net reclassification improvement (NRI), and integrated discrimination improvement (IDI) were calculated by risk assessment plot to compare the discriminative ability among models with and without radial artery pulse wave signals. After adjusting related confounding factors, radial artery pulse wave variable *h*_3_/*h*_1_ was selected as the sensitive influential factor for blood pressure. Moreover, a new model with *h*_3_/*h*_1_ had a higher AUC than the reference model without it (0.86 vs 0.84; *P*=0.030). And the NRI and IDI for the new model was 50.0% (*P*=0.017) and 3.16% (*P*=0.044), respectively. In addition to Ba-PWV, we found that the decrease of *t*_4_, *t*_5_, and *h*_5_ might be associated with higher FPG, TC, LDL-C, and UA and lower HDL-C. This research might provide a valuable additional tool for remote wearable monitoring of radial artery pulse wave signals in hypertension risk evaluation and management.

## 1. Introduction

Hypertension, as an important risk factor responsible for 19% of global deaths [1] and more than half of the development of a variety of cardiovascular disorders [2, 3], has become a growing global health issue. In particular, in the setting of an aging population such as China, according to the latest nationwide survey (2012–2015), the prevalence of hypertension among Chinese adults has reached 23.2%, while the awareness rate, treatment rate, and control rate are only 46.9%, 40.7%, and 15.3%, respectively [4]. Therefore, it is necessary to better understand risk factors for the development of hypertension, so as to facilitate optimal disease prevention and treatment strategies. In addition, high blood pressure is usually combined with other cardiovascular risk factors (obesity, dyslipidemia, and high blood glucose). It was reported that, in different components of the metabolic syndrome and its combination form, all the top seven cardiovascular risk factors had the characteristics of “abdominal obesity + high blood pressure”; in particular, in hypertension with abdominal obesity and low HDL-C, the cardiovascular risk is the highest (5.25 times) [5, 6].

From the perspective of modern medicine, arterial vessel is one of the main target organs in hypertension injury. Initially, its structural changes are adaptive, but it becomes maladaptive in chronic hypertension, leading to vascular remodeling and arterial stiffness [7, 8]. In particular, for arterial stiffness, it is one of the earliest recognized markers of hypertension and plays an important role in the development and prognosis of hypertension. It is reported that arterial stiffness makes an important contribution to the increase in systolic blood pressure (SBP) and the development of hypertension in general population, independently of traditional hypertension risk factors [9–15]. Besides, changes in both small and large arteries could predict future hypertension. It has also been recognized that narrowing of the retinal arteries is a marker of increased resistance in the microvasculature and that this can be detected before hypertension's development [16, 17]. Furthermore, the combination of arterial stiffness measured by the cardio-ankle vascular index (CAVI) and small artery retinopathy detected by retinal fundus photography could predict the development of hypertension [18].

Although from the perspective of traditional Chinese Medicine (TCM), as we know, hypertension belongs to “vertigo” or “headache,” the main disease location in hypertension is liver, just like the saying in Huang Di's Canon of Internal Medicine: “all wind and dizziness disorders belong to the liver.” According to the TCM theory, liver controls the function of storing blood; that is to say, the liver is capable of retaining blood and regulating the amount of blood in the body. However, in TCM, it is also said that the pulse is the mansion of blood; therefore, we speculate that the pulse diagnosis might play a role in hypertension prediction. The pulse state of radial artery, as one part of the arterial system, has been described in traditional Chinese medicine (TCM) and other related oriental medicine systems. In order to overcome the subjectivity and ambiguity of the traditional description, considerable researches on varying pulse waveform acquisition sensors [19–21], pulse waveform preprocessing, and feature extraction [22–24] have been carried out recently to measure radial pulse objectively and automatically. Further, the radial artery pulse wave of hypertension has been studied. It was reported that the maximum pulse amplitude of radial artery pulse wave in hypertension group was higher than those of the healthy group and was associated with hypertension independently of age and BMI [25, 26]. In addition, the association of pulse image analysis (PIA) with pulse wave analysis (PWA) and hemodynamics in patients with hypertension was evaluated, and it revealed that high values of pulse wave velocity and blood pressure were related to superficial, strong, and fast pulse images [27]. On the other hand, the parameters of both PWA and PIA were used to identify patterns in patients with hypertension and the associations of those patterns with functional capacity measured by handgrip strength and physical activity [28]. Besides, the characteristics of pulse wave variables in hypertensive patients and healthy subjects can be applied in the diagnosis of Sasang constitution [25] and were associated with the quality of life [29]. Moreover, we have previously studied cardiovascular pathophysiological mechanism of radial artery pulse wave variables in hypertension. The results suggested that radial artery pulse wave variables are actually a comprehensive reflection of both arterial stiffness and cardiovascular functions. The decrease of *t*_1_, *t*_5_, *t*_3_, *h*_5_ and increase of *h*_1_, *h*_3_/*h*_1_ were independently associated with arterial stiffness, while the height of dicrotic wave indicated by *h*_5_ was the most relevant pulse wave variable to the echocardiographic parameters [30]. Furthermore, the radial artery pulse wave variables were applied to predict the development of hypertension by varying machine learning methods (ANN, AdaBoost, Gradient Boosting, and Random Forest, etc.), and they achieved an accuracy of about 80% [31–33].

Thus, both arterial stiffness and radial artery pulse wave parameters could predict hypertension. However, it is not yet clear whether the BP-increasing effects of arterial stiffness and radial artery pulse wave parameters are related. No study has investigated the role of radial pulse wave signals combined with Ba-PWV in the prediction of hypertension and the monitoring of its concomitant metabolic risk factors in a hypertensive population. The purpose of this study was to investigate whether radial pulse wave variables improve the risk prediction of incident hypertension beyond traditional cardiovascular risk factor Ba-PWV, and detect the association of metabolic risk factors with Ba-PWV and radial artery pulse wave parameters, which might provide a probability for remote wearable monitoring of radial artery pulse wave variables in the hypertension development and management.

## 2. Methods

### 2.1. Study Population

523 Chinese subjects were enrolled in the study from April 2016 to March 2018, and they were all from the First People's Hospital of Taicang affiliated to Suzhou University. Inclusion criteria were complete data on brachial-ankle pulse wave velocity (Ba-PWV), complete data on radial artery pulse wave variables, and the related metabolic blood biochemical indicator data. The recruited participants were divided into two groups: normotensive group and hypertensive group. According to the Chinese guideline for the prevention and management of hypertension (revised edition in 2018) [34], the inclusion criteria were as follows: patients with confirmed diagnosis of hypertension; normotensive patients but with administration of antihypertensive medication. Hypertension was defined as systolic blood pressure ≥140 mm·Hg or diastolic blood pressure ≥90 mm·Hg when detected under the static state and during three visits of different dates.

The Medical Ethics Committee of the First People's Hospital of Taicang affiliated to Suzhou University approved the study, and written informed consent was obtained from all included subjects according to the Declaration of Helsinki.

### 2.2. Data Collection

Patients' metadata were collected (i.e., sex, age, BMI, duration time of hypertension, history of taking antihypertensive drug, and the controlling condition of blood pressure). Physical examinations included blood pressure, pulse pressure, brachial-ankle pulse wave velocity (Ba-PWV), and blood biochemical indicators for metabolic risk factors data. In this study, blood pressure and Ba-PWV were measured via the commercially available digital automatic BP monitor and arteriosclerosis detector (OMRON model, BP-203RPEIII, Japan).

### 2.3. Radial Artery Pulse Recordings and Analysis

**Figure 1:**
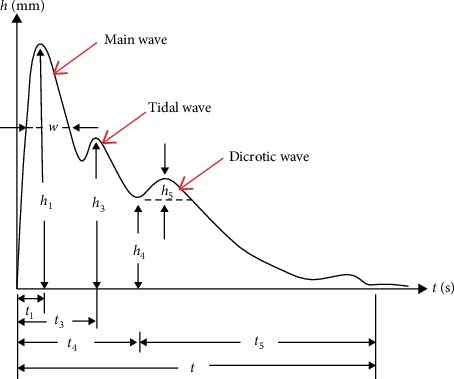
The time-domain variables of pulse signal. Notes. This pulse signal sample is a triple-peak waveform, which includes the main wave, the tidal wave, and the dicrotic wave. The *y*-axis is the amplitude of the pulse signal whose unit is millimeter (mm). The *x*-axis is the time whose unit is second(s).

By using a DDMX-100 type pulse measurement device developed by Shanghai University of Traditional Chinese Medicine, radial artery pulse images on the left “Guan” pulse position were recorded. Here, we only choose the left “Guan” pulse position for the following two reasons: one is that “Guan” pulse is the strongest pulse fluctuation point among the three parts of pulse, and the other is to be consistent with our previous study on this field [30] and form a unified research standard. The whole manipulation was conducted by two trained practitioners in a quiet and relaxed condition; the detailed procedure was mentioned in our previous study [30]. All the pulse waves we collected are from the period of 8:00 to 10:00 in the morning. Besides, in order to minimize the effect of blood pressure's changes over time on pulse wave as much as possible, we recorded the pulse wave instantly after we finished the collection of both blood pressure and Ba-PWV. Then, the most commonly used pulse wave form analysis in TCM—the classical time-domain variables *h*_1_, *h*_4_, *h*_5_, *t*_1_, *t*_3_, *t*_4_, *t*_5_, *h*_3_/*h*_1_, *h*_4_/*h*_1_, *w*/*t*—was performed using the software included in the DDMX-100 type pulse measurement device (see Figure 1) [35, 36].

### 2.4. Statistical Analysis

Unless otherwise stated, data are presented as mean ± SD or proportions (in percentages). In the continuous data, *t*-test or Mann–Whitney *U* test was used to compare differences between two groups, respectively, for the normally or non-normally distributed data. In the categorical data, Pearson *x*^2^ test was used to compare differences between two groups.

To identify the association between radial pulse variables and blood pressure, linear and stepwise regression analysis was performed using two different models. Model 1 was adjusted for age, sex, and BMI. Model 2 included model 1 and was additionally adjusted for duration time of hypertension, history of taking antihypertensive drug, and controlling condition of blood pressure.

To compare the discriminative ability among models with and without radial artery pulse wave variables, the area under the receiver-operating characteristic curve (AUC), net reclassification improvement (NRI), and integrated discrimination improvement (IDI) were calculated. The reference model included only age, sex, BMI, and Ba-PWV, whereas the new model included reference model and radial pulse wave variables. Furthermore, the potential additional value of radial pulse wave variables was assessed by the risk assessment plot. This is a popular method to assess the performance/usefulness of a new biomarker by proposing two new statistical indicators, namely, NRI and IDI, in addition to the classical AUC. And this risk model is based on Cox proportional hazards for time-to-event data and logistic regression for case-control data. As this a case-control study, the logistic regression models were employed with sex as dichotomous and age, BMI, Ba-PWV, and radial pulse wave variables (in one of the two models) as continuous predictors. The outcomes of the models are dichotomous (hypertension or not). The AUC was a summary measure for discrimination between individuals who developed hypertension and those who did not. NRI focuses on reclassification tables constructed separately for participants with and without events and quantifies the correct movement in categories—upwards for events and downwards for nonevents—when adding the new risk factor [37]. IDI measures how the *R*^2^ (explained variance) improves with the addition of the new risk factor [38]. Because no established net reclassification improvement categories exist to guide clinical decisions for hypertension risk in Chinese adults, we only calculated continuous NRI.

All statistical analyses were performed with IBM SPSS 21.0 (IBM Corporation, Armonk, NY, USA), and the risk assessment plot was conducted in Matlab R2016a. All reported *P* values were 2-tailed, and those <0.05 were considered statistically significant.

## 3. Results

### 3.1. Baseline Characteristics of the Study Subjects

The baseline characteristics of 523 participants are shown in Table 1. In the study cohort, 264 subjects met the hypertension criteria; 259 subjects were in the normotensive group.

Higher BMI, systolic blood pressure, diastolic blood pressure, pulse pressure, FPG, TG, UA, Ba-PWV, and proportions of male but lower HDL-C values were observed in the hypertensive group than in the normotensive group (*P* < 0.05). As for the radial artery pulse wave variables, *t*_3_ and *t*_5_ of hypertensive group were lower than those of the normotensive group, while *h*_3_/*h*_1_ and *w*/*t* of hypertensive group were higher than those of the normotensive group (*P* < 0.05).

### 3.2. The Association between Radial Artery Pulse Wave Variables and Blood Pressure

The association between radial artery pulse wave variables and blood pressure in the total studied population is shown in Table 2. Stepwise regression analysis using model 1 demonstrated that systolic blood pressure (SBP) was independently associated with *h*_3_/*h*_1_ value (*β* = 0.30, *P* < 0.001), *h*_1_ value (*β* = 0.23, *P* < 0.001), *t*_3_ value (*β* = −0.16, *P* < 0.001), and *t*_5_ value (*β* = −0.12, *P*=0.02). Diastolic blood pressure (DBP) was independently associated with *t*_3_ value (*β* = −0.38, *P* < 0.001), *w*/*t* value (*β* = 0.24, *P* < 0.001), *t*_1_ value (*β* = 0.23, *P* < 0.001), and *h*_1_ value (*β* = 0.12, *P*=0.01). Pulse pressure (PP) was independently associated with *h*_1_ value (*β* = 0.30, *P* < 0.001), *h*_3_/*h*_1_ value (*β* = 0.20, *P* < 0.001), and *t*_1_ value (*β* = −0.13, *P* < 0.001).

Besides, after additional adjustment for duration time of hypertension, history of taking antihypertensive drugs, and controlling condition of blood pressure (model 2), systolic blood pressure (SBP) was still independently associated with *h*_3_/*h*_1_ value (*β* = 0.21, *P* < 0.001), *h*_1_ value (*β* = 0.18, *P* < 0.001), *t*_3_ value (*β* = −0.13, *P* < 0.001), and *t*_5_ value (*β* = −0.10, *P*=0.03), and so did pulse pressure (PP) with *h*_1_ value (*β* = 0.25, *P* < 0.001), *h*_3_/*h*_1_ value (*β* = 0.16, *P* < 0.001), and *t*_1_ value (*β* = −0.12, *P*=0.02). However, diastolic blood pressure (DBP) is only independently associated with *t*_5_ value (*β* = −0.22, *P* < 0.001) and *h*_3_/*h*_1_ value (*β* = 0.20, *P* < 0.001).

The above results suggested that higher systolic blood pressure (SBP) was independently correlated with higher *h*_3_/*h*_1_ and *h*_1_ value but lower *t*_3_ and *t*_5_ value. Diastolic blood pressure (DBP) was positively associated with *h*_3_/*h*_1_ value but negatively associated with *t*_5_ value independently of the above adjusted risk factors. For pulse pressure (PP), *h*_1_ value, *h*_3_/*h*_1_ value, and *t*_1_ value were its independent factors. In one word, among all the pulse wave variables, the common influential factor for SBP, DBP, and PP was *h*_3_/*h*_1_ value, which suggested that *h*_3_/*h*_1_ value was the most related pulse wave variable to blood pressure.

### 3.3. Comparison of Models with/without Radial Artery Pulse Wave Variables for the Prediction of Hypertension

**Figure 2:**
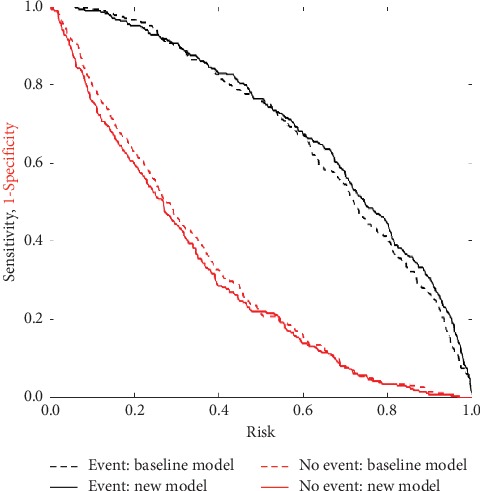
Additional value of radial artery pulse wave parameters compared with the baseline model for the prediction of hypertension. Notes. Risk assessment plot shows the baseline model (dashed lines) and the new model including _3_/_1_ (solid lines). Event curves (black lines) represent sensitivity vs calculated risk. No event curves (red lines) represent 1 − specificity vs calculated risk. Baseline model: age, sex, body mass index (BMI), and Ba-PWV. New model: baseline model + *h*_3_/*h*_1_.

Two models with/without radial artery pulse wave variables were compared for their ability to classify participants into the normotensive and hypertensive categories (Table 3). The reference model included age, sex, BMI, and Ba-PWV. In the association result between radial artery pulse wave variables and blood pressure, *h*_3_/*h*_1_ value was the only related pulse wave variable to systolic blood pressure, diastolic blood pressure, and pulse pressure; therefore, the new model additionally included radial artery pulse wave variable *h*_3_/*h*_1_. The new model had a higher area under the receiver-operating characteristic curve (0.86) than the reference model (0.84; *P*=0.030). The continuous net reclassification improvement for the new model was 50.0% (*P*=0.017). The integrated discrimination improvement for the new model was 3.16% (*P*=0.044). The risk assessment plot supported the additional value of radial artery pulse wave variable *h*_3_/*h*_1_ in the incident hypertension risk assessment (Figure 2).

### 3.4. The Association of Radial Artery Pulse Wave Variables and Ba-PWV with Other Metabolic Risk Factors

All the six metabolic risk factors, after adjusting for age, sex, BMI, blood pressure, pulse pressure, duration time of hypertension, history of taking antihypertensive drugs, and controlling condition of blood pressure, are independently associated with radial artery pulse wave variables/Ba-PWV.  Table 4 shows that *t*_5_ was the most related indicator to metabolic risk factors like FPG (*β* = −0.19, *P*=0.001), TC (*β* = −0.12, *P*=0.034), and LDL-C (*β* = −0.14, *P*=0.016); next was Ba-PWV with FPG (*β* = 0.24, *P*=0.008) and TG (*β* = 0.31, *P*=0.001), while *t*_4_ and *h*_5_ were the only independent factors for HDL-C (*β* = 0.13, *P*=0.013) and UA (*β* = −0.14, *P*=0.014), respectively.

The above results indicate that arterial stiffness is more related to FPG and TG, while the pulse wave variables such as the decrease of the systole period of the left ventricle (*t*_4_), the diastolic period of the left ventricle (*t*_5_), and the height of the dicrotic wave (*h*_5_) were associated with higher FPG, TC, LDL-C, and UA and lower HDL-C.

## 4. Discussion

The present study examined the effects of radial pulse wave variables on risk prediction of hypertension among Chinese adults and produced three main findings. First, radial pulse wave variable *h*_3_/*h*_1_ was the common indicator which is directly and independently associated with systolic blood pressure, diastolic blood pressure, and pulse pressure. Second, radial pulse wave variable *h*_3_/*h*_1_ improved the incident hypertension risk prediction beyond traditional cardiovascular risk factor Ba-PWV. Third, other radial pulse wave variables like *t*_4_, *t*_5_, and *h*_5_ might be associated with higher FPG, TC, LDL-C, and UA and lower HDL-C independently of other confounding risk factors in a general hypertensive population.

We conducted this study on the basis that various researches have verified the role of arterial stiffness as a risk factor for clinical hypertension. For the measurements of arterial stiffness, several different commercially available devices were used at several anatomical sites, including carotid-radial, femoral-tibial, and carotid-femoral arterial segments as well as brachial-ankle or single-point pulse wave velocity measurements [39]. Besides, the acknowledged gold standard method for assessing arterial stiffness is carotid-femoral pulse wave velocity for the reason that it is a direct reflection of arterial stiffness and it has the best predictive value for cardiovascular outcome [40, 41]. However, in this study, we used the Ba-PWV rather than carotid-femoral pulse wave velocity to measure arterial stiffness for it can prevent the groin area exposure which occurred in the collection of carotid-femoral PWV and is therefore more often used in Asian countries [42].

The findings show a link between blood pressure and radial pulse wave variables, which is basically consistent with existing literature [27, 30]. Table 2 shows that higher *h*_1_, *h*_3_/*h*_1_ and shorter *t*_1_, *t*_3_, *t*_5_ values were associated with higher SBP, DBP, and PP. However, in our previous study on the association between radial pulse wave variables and Ba-PWV, we found that the increased Ba-PWV was independently related to higher *h*_1_, *h*_3_/*h*_1_ and lower *t*_1_, *t*_3_, *t*_5_, *h*_5_ values. As a result, we can conclude that blood pressure, arterial stiffness, and radial pulse wave variables are mutually correlated to each other.

By identifying the radial pulse wave variable *h*_3_/*h*_1_ as a common influential factor for SBP, DBP, and PP, we also investigated its role in the risk prediction of hypertension. We have shown for the first time that radial pulse wave variable *h*_3_/*h*_1_, which was significantly augmented in hypertension patients and associated with all blood pressure indicators (SBP, DBP, and PP), is an independent additive predictor of incident hypertension beyond the traditional cardiovascular risk factor Ba-PWV. The radial artery pulse wave variable *h*_3_/*h*_1_, which represents the amplitude ratio of tidal wave to main wave, can reflect the intensity of reflection wave [43]. In addition, it was reported that *h*_3_/*h*_1_ was selected as one of the four important features to classify the pulse image (the sliding vein, chord vein, and chord pulse) of TCM by application of Gradient Boosting Decision Tree (GBDT) along with the other radial artery pulse wave variables, *h*_4_/*h*_1_, *w*/*t*, and Rf [44]. Furthermore, Su et al. [45] studied the arterial waveform changes by local cold stimulation to induced anomalous pulse waveform for arterial stiffness at three stages (base, cold, and recovery), and they found that *h*_3_/*h*_1_ was significantly higher at cold than at base stage, which indicates that *h*_3_/*h*_1_ might be an important indicator of the arterial stiffness of radial artery. Unlike Ba-PWV, which is actually the direct reflection of arterial stiffness of the brachial-ankle arterial segments by calculating the pulse wave velocity, the pulse wave parameters we used in this study are actually time-domain features extracted from the pressure wave of the radial artery. As for the relationship between them, they both can reflect the arterial stiffness. However, based on our previous study [30], the main difference between them is that radial arterial pulse parameters are a comprehensive manifestation of both arterial elasticity and cardiovascular function. In addition, the arterial stiffness they reflect come from different arterial segments. This might explain why the addition of radial artery pulse wave parameter *h*_3_/*h*_1_ can improve the risk prediction of hypertension.

In the clinical setting, high blood pressure usually coexists with other cardiovascular risk factors (obesity, dyslipidemia, and high blood glucose), for example, in the form of metabolic syndrome, which will increase the cardiovascular risk. Therefore, we further preliminarily detected the association of other metabolic risk factors (FPG, TC, TG, HDL-C, LDL-C, and UA) with Ba-PWV and radial artery pulse wave variables in this study. And we found that the decreased changes of systole period of the left ventricle (*t*_4_), the diastolic period of the left ventricle (*t*_5_), and the height of the dicrotic wave (*h*_5_) were especially associated with higher FPG, TC, LDL-C, and UA and lower HDL-C. These results also indicated that radial artery pulse wave signals might be one kind of supplementary indicators beyond Ba-PWV to manifest the above abnormal metabolic risk factors. It is reported that pathophysiological changes in the arterial vascular system occurring in the metabolic syndrome might be an important factor which increases the cardiovascular risk. And the changes of arterial vascular system involve both macrovascular adaptions (conduit remodeling and large artery endothelial dysfunction) and microvascular adaptions (microvascular remodeling, microvascular rarefaction, and compromised vasodilation) [46]. These might be the possible mechanisms through which both radial artery pulse wave signals and Ba-PWV are correlated with metabolic risk factors, like FPG, TC, LDL-C, UA, and HDL-C.

To the best of our knowledge, this is the first retrospective study to show that radial artery pulse wave parameter *h*_3_/*h*_1_ is a significant predictor of hypertension. In addition, we found that both Ba-PWV and radial artery pulse wave parameters were associated with the combined metabolic risk factors independently of other confounding risk factors in a general hypertensive population. This study would provide an experimental basis for application of remote wearable monitoring of hypertension risk evaluation and management. The wearable devices based on radial artery pulse wave can be developed to provide remote dynamic monitoring, which mainly includes timely data monitoring and risk assessment. By integrating the timeliness, accessibility, and individuality of Internet technology, an accurate management system can be created for the prevention, monitoring, intervention, and protection of the hypertensive patients. In the end, it can help the primary care doctors to predict the disease change and take timely treatment measures to prevent the exacerbation of the disease, thus realizing individualized treatment for patients.

Our study has some limitations. A major limitation of the present study is that we only measured clinical BP rather than ambulatory or home blood pressure, which is unable to identify masked hypertension at baseline and white-coat hypertension at follow-up. In addition, as this was a cross-sectional study, we only found that radial artery pulse wave variable *h*_3_/*h*_1_ might improve the incident hypertension risk prediction beyond the already-known risk factor Ba-PWV. Further longitudinal studies should be conducted to determine whether these changes of radial artery pulse waves evolve in the clinical development of hypertension. Finally, in this retrospective study, we only investigate the history of taking antihypertensive drugs rather than the concrete type of the current antihypertensive drugs. More prospective studies are needed to identify the impact of different concrete types of the current antihypertensive drugs on the radial pulse wave changes.

## 5. Conclusion

Our current findings suggest that radial artery pulse wave variable *h*_3_/*h*_1_ is directly and independently associated with systolic blood pressure, diastolic blood pressure, and pulse pressure and might be related to the occurrence of hypertension in addition to the traditional Ba-PWV. Moreover, radial artery pulse wave variables *t*_4_, *t*_5_, *h*_5_ are independently related to its concomitant metabolic risk factors. Therefore, radial artery pulse wave signals might provide a valuable additional tool for remote wearable monitoring in hypertension risk evaluation and management.

## Figures and Tables

**Table 1:** Characteristics of the study subjects.

Variables	Normotensive group (*n* = 259)	Hypertensive group (*n* = 264)	*P* value
*Demographic factors*			
Age in years	50.98 ± 12.93	50.7 ± 12.36	0.649
Sex (male)	66.00%	75.40%	0.019
BMI (kg/m^2^)	24.41 ± 3.28	26.61 ± 3.6	<0.001
Systolic blood pressure (mmHg)	125.12 ± 10.94	151.94 ± 12.15	<0.001
Diastolic blood pressure (mmHg)	75.2 ± 8.7	92.8 ± 9.36	<0.001
Pulse pressure (mmHg)	49.92 ± 7.62	59.14 ± 11.82	<0.001

*Other metabolic risk factors*			
FPG (mmol/l)	5.63 ± 0.98	6.14 ± 1.66	<0.001
TC (mmol/l)	5.06 ± 1.07	5.1 ± 0.92	0.707
TG (mmol/l)	1.49 ± 0.85	2.02 ± 1.38	<0.001
HDL-C (mmol/l)	1.25 ± 0.26	1.15 ± 0.28	<0.001
LDL-C (mmol/l)	3.11 ± 0.93	3.05 ± 0.74	0.748
UA (mmol/l)	359.15 ± 90.31	375.81 ± 83.6	0.034

*Arterial stiffness measurements*			
Ba-PWV (cm/s)	1424.84 ± 222.64	1698.69 ± 333.52	<0.001
ABI	1.09 ± 0.08	1.09 ± 0.08	0.561

*Radial artery pulse variables*			
*t*_1_ (s)	0.13 ± 0.03	0.13 ± 0.03	0.570
*t*_3_ (s)	0.25 ± 0.03	0.24 ± 0.03	<0.001
*t*_4_ (s)	0.33 ± 0.03	0.33 ± 0.04	0.474
*t*_5_ (s)	0.49 ± 0.11	0.47 ± 0.11	0.004
*h*_1_ (mm)	13.23 ± 4.53	14.06 ± 5.28	0.144
*h*_5_ (mm)	−0.11 ± 0.52	-0.19 ± 0.75	0.906
*h*_3_/*h*_1_	0.75 ± 0.14	0.82 ± 0.16	<0.001
*h*_4_/*h*_1_	0.45 ± 0.1	0.46 ± 0.11	0.460
*w*/*t*	0.23 ± 0.04	0.24 ± 0.04	<0.001

Data shown are mean ± SD or proportions (in percentages). FPG: fasting plasma glucose; TC: total cholesterol; TG: triglyceride; HDL-C: high-density lipoprotein cholesterol; LDL-C: low-density lipoprotein cholesterol; UA: uric acid.

**Table 2:** The association between blood pressure and radial artery pulse in the total studied population.

Dependent	Variables entered	*R* ^2^	*B*	95% CI of B	*β*	*P* value
SBP	Model 1					
	*h*_3_/*h*_1_	0.38	34.15	[22.96, 45.34]	0.30	＜0.001
	*h*_1_		0.95	[0.58, 1.32]	0.23	＜0.001
	*t*_3_		−94.47	[−151.51, −37.43]	−0.16	＜0.001
	*t*_5_		−22.09	[−40.36, −3.83]	−0.12	0.02
	Model 2					
	*t*_3_	0.54	−76.39	[−127.24, −25.53]	−0.13	＜0.001
	*h*_1_		0.74	[0.40, 1.08]	0.18	＜0.001
	*h*_3_/*h*_1_		24.34	[13.97, 34.72]	0.21	＜0.001
	*t*_5_		−18.51	[−35.13, −1.89]	−0.10	0.03

DBP	Model 1					
	*t*_3_		−160.71	[−216.27, −105.14]	−0.38	＜0.001
	w/*t*		74.67	[45.38, 103.97]	0.24	＜0.001
	*t*_1_	0.37	106.03	[41.82, 170.23]	0.23	＜0.001
	*h*_1_		0.34	[0.07, 0.62]	0.12	0.01
	Model 2					
	*t*_5_	0.5	−28.31	[−39.62, −17.00]	−0.22	＜0.001
	*h*_3_/*h*_1_		16.39	[8.85, 23.92]	0.20	＜0.001

Pp	Model 1					
	*h*_1_		0.66	[0.43, 0.89]	0.30	＜0.001
	*h*_3_/*h*_1_		12.13	[5.79, 18.46]	0.20	＜0.001
	*t*_1_	0.22	−45.85	[−83.06, −8.65]	−0.13	＜0.001
	Model 2					
	*h*_1_	0.29	0.54	[0.31, 0.77]	0.25	＜0.001
	*h*_3_/*h*_1_		10.12	[3.71, 16.54]	0.16	＜0.001
	*t*_1_		−44.7	[−81.42, −7.98]	−0.12	0.02

Stepwise regression included the radial artery pulse wave variables; age, sex, and BMI (model 1); and, additionally, use of duration time of hypertension, history of taking antihypertensive drugs, and the controlling condition of blood pressure (model 2). The partial regression coefficient B, 95% CI of B, and standard regression coefficient *β* are shown for parameters entered into the model.

**Table 3:** Comparison of models with/without radial artery pulse wave variables for the prediction of hypertension.

Model	AUC	95% CI	*P* value	NRI events (%)	NRI nonevents (%)	NRI total (%)	*P* value	IDI events (%)	IDI nonevents (%)	IDI total (%)	*P* value
Baseline model	0.84	0.81–0.88	…	…	…	…	…	…	…	…	…
New model	0.86	0.83–0.89	0.030	28.0	22.0	50.0	0.017	1.57	1.60	3.16	0.044

Baseline model: age, sex, BMI, and Ba-PWV. New model: baseline model + *h*_3_/*h*_1_. Hypertension was defined as systolic blood pressure ≥140 mm·Hg or diastolic blood pressure ≥90 mm·Hg. AUC: area under the receiver-operating characteristic curve; CI: confidence interval; event: incident hypertension; NRI: continuous net reclassification improvement; IDI: integrated discrimination improvement; PWV: pulse wave velocity. *h*_3_/*h*_1_: the ratio of the tidal wave height (*h*_3_) to the main wave height (*h*_1_).

**Table 4:** The association between other cardiovascular metabolic risk factors and radial artery pulse/Ba-PWV.

Dependent	Variables entered	*R* ^2^	*B*	95% CI of *B*	*β*	*P* value
FPG	*t*_5_ (s)	0.20	−2.767	[−4.326, −1.209]	−0.19	0.001
	Ba-PWV (cm/s)		0.001	[0.000, 0.002]	0.24	0.008
TG	Ba-PWV (cm/s)	0.15	0.001	[0.001, 0.002]	0.31	0.001
TC	*t*_5_ (s)	0.05	−1.195	[−2.300, −0.091]	−0.12	0.034
HDL-C	*t*_4_ (s)	0.22	1.025	[0.219, 1.832]	0.13	0.013
LDL-C	*t*_5_ (s)	0.04	−1.131	[−2.051, −0.211]	−0.14	0.016
UA	*h*_5_ (mm)	0.24	−16.184	[−29.113, −3.256]	−0.14	0.014

Stepwise regression was used by adjusting for age, sex, BMI, blood pressure, pulse pressure, duration time of hypertension, history of taking antihypertensive drugs, and the controlling condition of blood pressure. The partial regression coefficient *B*, 95% CI of *B*, and standard regression coefficient *β* are shown for parameters entered into the model. FPG: fasting plasma glucose; TC: total cholesterol; TG: triglyceride; HDL-C: high-density lipoprotein cholesterol; LDL-C: low-density lipoprotein cholesterol; UA: uric acid.

## Data Availability

The datasets generated and analyzed during the current study are not publicly available due to the confidentiality of the data, which is an important component of the National Key Technology R&D Program of the 13th Five-Year Plan (No. 2017YFC1703301) in China, but are available from the corresponding author on reasonable request.
